# Long-term colonisation with donor bacteriophages following successful faecal microbial transplantation

**DOI:** 10.1186/s40168-018-0598-x

**Published:** 2018-12-10

**Authors:** L. A. Draper, F. J. Ryan, M. K. Smith, J. Jalanka, E. Mattila, P. A. Arkkila, R. P. Ross, R. Satokari, C. Hill

**Affiliations:** 1 APC Microbiome Institute, University College Cork, Cork, Ireland; 2 Present Address: South Australian Health and Medical Research Institute, North Terrace, Adelaide, 5000 Australia; 3 Immunobiology Research Program and Department of Bacteriology and Immunology, Faculty of Medicine, University of Helsinki, Helsinki, Finland; 4 Departments of Gastroenterology and Infectious Diseases, Helsinki University Hospital, Helsinki, Finland

**Keywords:** Faecal microbiota transplantation, Bacteriophages, Viruses, Engraftment, Persistence, Donor-recipient

## Abstract

**Background:**

Faecal microbiota transplantation (FMT) is used in the treatment of recurrent *Clostridium difficile* infection. Its success is typically attributed to the restoration of a diverse microbiota. Viruses (including bacteriophages) are the most numerically dominant and potentially the most diverse members of the microbiota, but their fate following FMT has not been well studied.

**Results:**

We studied viral transfer following FMT from 3 donors to 14 patients. Recipient viromes resembled those of their donors for up to 12 months. Tracking individual bacteriophage colonisation revealed that engraftment of individual bacteriophages was dependent on specific donor-recipient pairings. Specifically, multiple recipients from a single donor displayed highly individualised virus colonisation patterns.

**Conclusions:**

The impact of viruses on long-term microbial dynamics is a factor that should be reviewed when considering FMT as a therapeutic option.

**Electronic supplementary material:**

The online version of this article (10.1186/s40168-018-0598-x) contains supplementary material, which is available to authorized users.

## Background

The human gut houses a densely populated microbial ecosystem that includes bacteria, archaea, protozoa, and fungi, as well as their viruses. These coexist in a dynamic but stable equilibrium in healthy individuals [1]. Disruption of this complex ecosystem has been associated with numerous diseases [2], for example antibiotic treatment can potentially lead to recurrent *Clostridium difficile* infection (rCDI). Faecal microbiota transplantation (FMT) involves the infusion of a faecal slurry from a healthy donor to the duodenum, caecum, or colon of a recipient in an attempt to restore microbiota diversity and composition. FMT is used in clinical practice and has a reported 80–90% success rate in treating rCDI [3]. It has been shown that bacterial transfer occurs in a donor-specific manner and that the new resident species can be retained for up to 1 year post-FMT [4]. Numerically, the most predominant component of the gut microbiome is bacteriophages, which may be 20 times more abundant than bacteria in mucosal samples [5, 6]. These bacteriophages play an essential role in population dynamics in aquatic environments and presumably have similar effects in the gut microbial ecosystem. To date, only limited investigations into the long-term transplantation of bacteriophages following FMT have been performed. These include two rCDI case studies spanning 6–7 months post-FMT [7, 8] and a ~ 3.5-month study following the unsuccessful FMT treatment of three paediatric ulcerative colitis patients [9].

Resolution of rCDI is the primary goal of FMT treatment, but in order to generate a more reproducible treatment regime, significant efforts have been devoted to identifying a more defined group of bacterial species that could replace FMT. Petrof et al. [10] succeeded in disease resolution for up to 6 months in 2 patients using a combination of 33 bacterial isolates recovered from a healthy donor stool sample. However, Ott et al. [11] have shown that in a small study involving 5 patients, a sterile faecal filtrate from which cellular microbes (but not bacteriophages) were removed also had the ability to resolve rCDI. Moreover, Zuo et al. [8] found that treatment response in FMT was associated with a high colonisation level of donor-derived *Caudovirales* taxa in the recipient and concluded that *Caudovirales* may play a role in the efficacy of FMT in rCDI. The current study is the first to investigate long-term engraftment of bacteriophages following successful FMT treatment of rCDI patents. The study includes 3 donors and 14 recipients from a recent study involving patients with rCDI [4], the majority of which were monitored for up to 1 year and reveals that the transfer and persistence of bacteriophages in the human gut are specific to each donor-recipient pair.

## Results and discussion

Shotgun metagenomics was used to analyse the viromes of these donors and recipients. This corresponded to 134 faecal samples collected for 14 recipient patients pre-FMT and at intervals up to 1-year post-FMT, as well as samples from each of the 3 donors collected at the corresponding time points. From 2 recipients (P11 and P14), no pre-FMT sample was available, while some post-FMT samples were unavailable for 3 recipients. Metadata corresponding to these donors and recipients can be found in Additional file 1: Table S1 and in the original study examining the bacteriome of these individuals [4]. Viral DNA was sequenced on an Illumina HiSeq to a mean depth of 1,277,374 paired-end reads per sample and assembled with metaSPAdes. In order to avoid contamination with bacterial DNA, confounding estimates of viral transfer, only those contigs that contained known viral genes or were predicted as viral with VirSorter [12] were used for further analysis resulting in a total of 7064 metagenomic viral contigs with a mean length of 4.9 kb containing an average of 5.7 known viral genes per contig. The bacteriophage community was dominated by dsDNA and ssDNA phages. The majority of RNA viruses found in the gut are plant and human viruses [13], and thus, these were not investigated here, as the current study focused on DNA containing viral particles of the virome. Nonetheless, the impact and involvement of eukaryote and other RNA viruses are of interest, especially as it has been previously reported that the eukaryotic virome in the gut is altered post-FMT in ulcerative colitis (UC) patients [14]. Whether this is UC-specific phenomenon or a general effect of FMT is yet to be established.

**Fig. 1:**
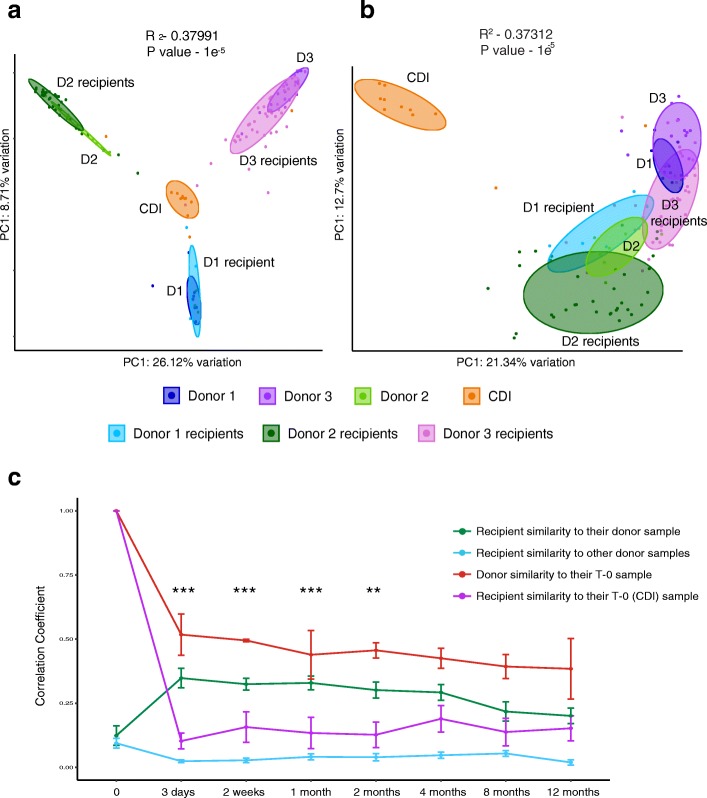
Viral profiles of donors and recipients. **a** Bray-Curtis PCoA plot based on virome abundance demonstrating that post-FMT recipients cluster strongly with their donor. **b** Bray-Curtis PCoA plot based on 16S abundance data demonstrating the bacterial genus level group clustering of donors and recipients. *P* value and *R*^2^ values were generated with PERMANOVA as implemented in the Adonis function in the Vegan package in R. **c** Plot shows similarity of individual recipient viromes at each time to themselves at the first time point, i.e., to their CDI sample (pink line), to their donor (green line), or to other donors (blue line) over time. Donor virome similarity to their original donated sample was also investigated over the 12-month period (red line). The *y*-axis values represent Spearman’s rho, with the mean and 95% confidence interval plotted per time point. Statistical significance, as assessed by the nonparametric Wilcoxon rank sum test, between the recipient’s similarity to their donor and recipient similarity to themselves pre-FMT is indicated by asterisks. *P ≤ 0.05, **P ≤ 0.01, ***P ≤ 0.001

The impact of FMT on the DNA virome beta diversity was measured using Bray-Curtis distance. Principal coordinate analyses of the virome demonstrated that FMT resulted in gut virome remodelling in a donor-specific fashion with each set of donor-recipient groups forming distinct groups centred on the donor (Fig. 1a), strikingly more distinct than that observed based on bacterial profiles as measured with 16S (Fig. 1b). In order to examine the level of donor-recipient similarity over time, we plotted the Spearman correlation between each recipient sample and its donor sample at each time point, the correlation between recipients and their own pre-FMT (rCDI) sample, and their correlation with other donors and finally donor similarity over time to their own T-0 sample (Fig. 1c). We found that for 2 months post-FMT, the recipient’s viromes were significantly more similar to that of their donors than it was to their own pre-FMT virome (Wilcoxon rank sum test, *P* = 0.01288). Moreover, for up to 12 months post-FMT, the recipients were found to be equally similar to their own pre-FMT sample and their donor’s sample. This demonstrates a lasting shift in the recipient gut virome towards a donor-specific one, and given that bacteriophages are usually strain-specific, this presumably reflects a long-term alteration to the fine structure of the microbiome.

**Fig. 2:**
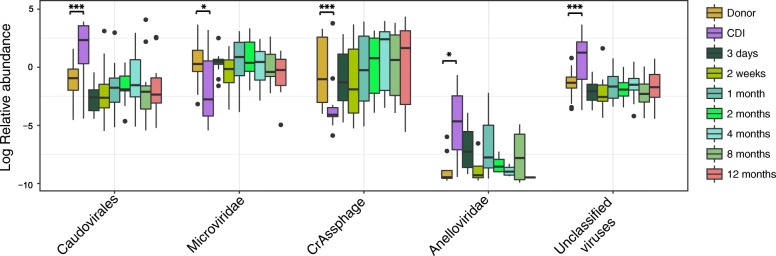
Abundances of viral taxonomic groups. **a** Boxplots showing relative abundances of viral taxonomic groups identified in this data by the Kaiju metagenomic classifier. Unclassified viruses refer to sequences classified as viral but not to any specific taxonomic group. Wilcoxon rank sum test was used to determine statistical differences in relative abundance between the donors and recipients pre-FMT and is indicated by asterisks; *P ≤ 0.05, **P ≤ 0.01, ***P ≤ 0.001. Whiskers indicate the highest or lowest occurring value within 1.5*IQR (interquartile range) of the upper or lower quartile

Recurrent CDI is marked by changes in the gut virome relative to healthy controls [8]. We investigated the abundance of viral taxonomic groups by utilising a read classification approach based on amino acid similarity to the Non-Redundant (nr) database at NCBI [15]. The vast majority of classifiable reads were identified as being from the *Caudovirales* and *Microviridae* taxa, indicating the samples were predominantly composed of bacteriophages as has been previously found with the human gut virome [16]. In agreement with Zuo et al., we found that rCDI is marked by a disturbance in the enteric gut virome characterised by increased *Caudovirales* (Wilcoxon test, *P* = 0.0009806), decreased *Microviridae* (Wilcoxon test, *P* = 0.01093), and increased *Anelloviridae* abundance (Wilcoxon test, *P* = 0.04509) when compared to our healthy donors. (Fig. 2). Furthermore, we have expanded upon previous observations by demonstrating that these differences are not only immediately resolved by FMT but persist for at least 12 months (Fig. 2). We also observed an increase in unclassified viruses (Wilcoxon test, *P* = 0.00039) in the rCDI virome and observed that these individuals have decreased levels of CrAssphage (Wilcoxon test, *P* = 0.0005647) (Fig. 2), which represents the most prominent group of bacteriophages in the human gut [17, 18]. This group of CrAssphage-like bacteriophage is thought to primarily predate on members of the phylum *Bacteroidetes*, which has previously been described as decreased in the CDI gut microbiota [19]. Jalanka et al. observed a 2.7-fold increase in the members of the *Bacteroidetes* phylum in this cohort related to *Bacteroides vulgatus* and *Prevotella oralis* using 16S rRNA profiling [2]. No single bacteriophage, virus, viral family, or viral contig could be detected as universally present post-FMT suggesting that no single bacteriophage or DNA virus was responsible for disease resolution. The only statistically significant difference in viral richness or diversity was observed with a reduction in Chao1 richness in samples collected at 2 weeks post-FMT when compared with the original CDI patient samples (paired Wilcoxon rank sum test, *P* = 0.0425, Additional file 2: Figure S1). This initial fluctuation in richness post-FMT was also observed by Zuo et al. [8], and we believe it may constitute an initial loss of unique donor/recipient phages as a consequence of loss of their bacterial host due to engraftment and shifting of other members of the bacterial population following FMT.

Chehound et al. [9] observed that certain temperate bacteriophages were transferred with greater efficiency than other members of the virome during FMT. We aimed to assess the prevalence of integrase genes within our viral contigs to determine the extent to which temperate phages were transferred in this study. Of the set of 7064 contigs assembled here, 916 could be classified into a viral order or family, and 182 of these were found to contain integrase genes. A further 486 contigs that could not be classified to a known viral group also contained a known integrase. Only 51 of these 1402 (916 + 486) were observed to be transferred from any donor to any recipient at any time point; however, it is possible many others were below the detection threshold. Thus, our analysis indicates that the collective virome contains at least several hundred temperate phages, and some of these are indeed transplanted. In addition to temperate phage transfer, we also observed the transplantation of lytic phages such as CrAssphage (deemed lytic as it contains no known integrase gene and has not been observed in bacterial genomes) and members of the predominantly lytic *Microviridae* family [20]. We believe this strongly indicates that transfer of lytic bacteriophages occurs during FMT and that these either transplanted with their host or acquired a new host within the recipient’s microbiome.

**Fig. 3:**
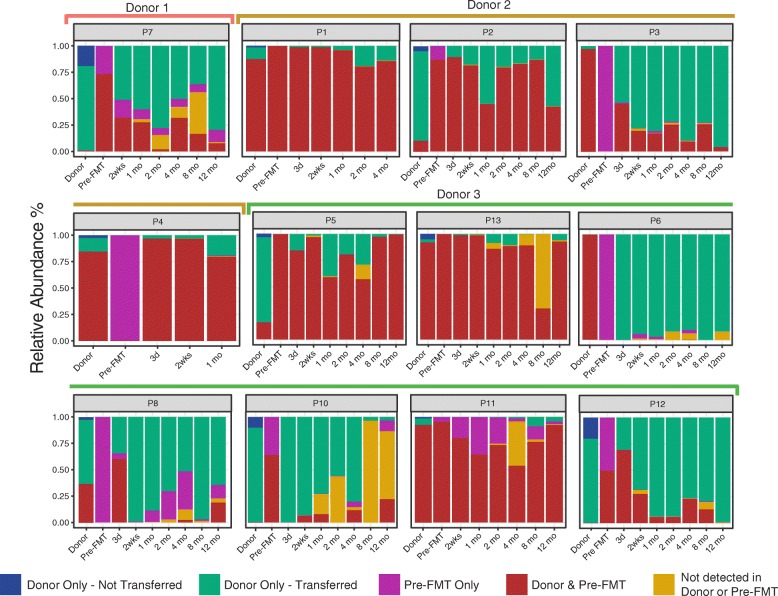
Relative viral abundances in donors and recipients. Relative abundance plots of donors per recipient and per time point with contig abundances summed by their origin, i.e., if they were transferred contigs unique to the donor (green), non-transplanted donor contigs (blue), unique recipient contigs (pink), contigs found in both donors and recipients (red), or contigs found in neither donors or pre-FMT at donation (yellow). In the case of two recipients (P11 and P14), pre-FMT samples were not available and so these individuals were omitted from this analysis

**Fig. 4:**
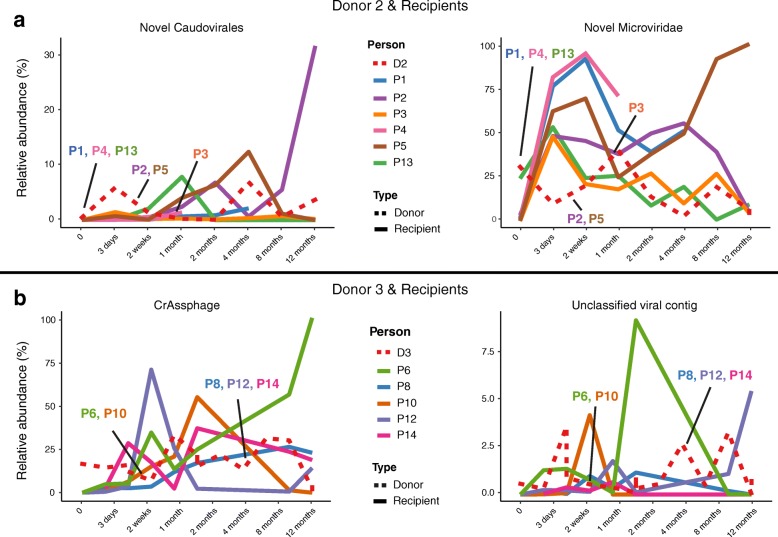
Relative abundance of individual bacteriophages in different donor-recipient pairs. **a** Line graphs representing relative abundance of different individual bacteriophages in the recipients of donor 2—a novel *Caudovirales* and *Microviridae*—and **b** recipients of donor 3—CrAssphage and an unclassified viral contig. A donor timeline depicting the relative abundance the bacteriophage in question has also been included in each case, with the time of donation to each recipient marked therein. As donor 1 only had a single recipient, it was not possible to determine if differential colonisation patterns were associated with this donor

Previous work has highlighted the differential colonisation of donor bacterial strains depending on the donor-recipient pairing post-FMT [4, 21–24]. Thus, we sought to explore viral colonisation across the 3 donors and 12 recipients (those with pre-FMT samples) for up to 1 year after treatment. Contig relative abundances were summed by origin (transferred and non-transferred contigs specific to the donor, unique recipient contigs, those common to both, or those newly detected post-FMT) (Fig. 3). We observed that the relative abundance of transferred contigs (those absent in CDI samples, present in the donor sample, and present post-FMT) is highly variable, ranging from almost the entire sample to a fraction of a percent depending on the donor-recipient pair. Individual’s viromes are highly unique [25]; thus, engraftment would be expected and is observed to be donor-specific (Fig. 1a). We therefore sought to investigate the colonisation patterns of specific bacteriophages. In all but one case, these phages were absent from the recipient and are examples of donor transfer but with differing recipient colonisation. A number of bacteriophages, including a member of the *Microviridae* family, a contig of 86 kb classified as a *Caudovirales*, CrAssphage, and an unclassified viral contig of 44 kb, none of which possess a known integrase, were transferred to all recipients but with varying degrees of engraftment and persistence over time (Fig. 4a, b). It is possible that the abundance of these lytic phages expands and contracts overtime due to fluctuations in host populations. Moreover, in some FMT recipients, high proportions of contigs were detected that were not detected in either CDI samples or in the donor. This suggests that these bacteriophages were either below the threshold of detection at the earlier time points, were the result of newly induced prophage, or were acquired exogenously by recipients.

Given that all recipients here achieved clinical resolution, it is impossible to ascertain what impact the colonisation of specific microbes played in the success of each FMT treatment. One plausible explanation is that a healthy, diverse, and stable ecosystem per se, even without specific or prescribed microbial components, can resolve rCDI. In such a scenario, bacteriophages could play a crucial role in maintaining ecosystem stability. Research to date has not established any difference in rCDI resolution following the use of related or unrelated healthy donor samples [26]. However, for large-scale implementation of FMT and especially for other indications, banks of screened faeces from healthy donors provide a more logistically sound approach. As highlighted here, and in previous studies [22], donor-recipient compatibility may be a key factor in the extent and stability of microbial colonisation. However, determination of donor-recipient microbiome similarity and/or compatibility in relation to disease resolution is still lacking. Virome analysis represents another view of the microbiome that could improve the selection of optimal donor-recipient pairs.

## Conclusion

The study of the role of the human microbiome on health and disease has largely focused on its bacterial component. A major success story has been the use of FMT for rCDI treatment in clinical practice. There have been conflicting reports in the literature as to what component of the microbiome is responsible for the resolution of rCDI, and the transfer of whole faecal material has proved most successful across studies. Similarly, the long-term effects of FMT on the microbiome and the virome of the recipient, and thus on human gastrointestinal health, are not well understood, with many studies following patients for less than half a year. Here, we demonstrate that the impact of a successful FMT on the virome lasts for 12 months and can result in the long-term colonisation of specific bacteriophages depending on the donor-recipient pairing. Bacteriophages are essential components of natural ecosystems and are likely to have an important stabilizing role in the gut ecosystem, too. We believe that those performing FMT therapy should consider all components of the microbiome when considering the optimal outcome and long-term health of their patients. Current donor eligibility screening is usually based on medical history, physical examination, and stool and blood screening [26–28]. Such screening with respect to donor viral content is suggested to identify the presence of major eukaryote viruses of note such as HIV, norovirus, adenovirus, rotavirus, and Epstein-Barr virus [26, 29], but this is not exhaustive nor does it pertain to bacteriophage content. Recent research has indicated that bacteriophages may play a role in gastrointestinal diseases such as IBD and in the development and maintenance of a healthy gut microbiome [30, 31], and thus, their transfer may have long-term consequences for human health. It is also worth noting, however, that up to 90% of sequences retrieved from a human virome are novel, or of unknown function [32], and so the transfer of a significant repertoire of unknown genetic information should also be considered when performing FMT for non-life-threatening diseases. However, given the practicality that full metagenomic/virome analysis is not feasible nor recommended in clinical practice at present [26], we believe that clinicians should continue, as they currently do so, to weigh the expected benefits and potential risks carefully when performing FMT, as, despite these current unknowns, FMT is a highly effective treatment for rCDI. Investigations into phageome/virome transfer in FMT should however continue in a research setting so as to understand more clearly the colonisation patterns and role of these dominant members of the microbiota.

## Materials and methods

### Sample collection and storage

Faecal samples from 14 recurrent *Clostridium difficile* infection (rCDI) patients and from 3 universal donors were utilised in this study. Samples from donors and patients were both pre- and post-FMT over a 1-year follow-up period (Additional file 1: Table S1). The study was approved by the Ethics Committee of the Hospital District of Helsinki and Uusimaa Finland (DnroHUS124/13/03/01/11) [4]. All samples were stored at − 80 °C prior to analysis.

### Virome DNA extraction and library preparation for MiSeq

Approximately 0.5 g of faecal sample was homogenised in 10 ml saline magnesium (SM) buffer (100 mM NaCl, 8 mM MgSO_4_, 50 mM Tris [pH 7.5]), followed by centrifugation twice at 5000*g* at 4 °C for 20 min. Resulting supernatants were filtered twice through 0.45 μm syringe filters to remove particulates and bacterial cells. NaCl (0.5 M final concentration; Sigma) and 10% *w*/*v* polyethylene glycol (PEG-8000; Sigma) were added to the resulting filtrates, and these were incubated at 4 °C overnight. Following centrifugation at 5000*g* at 4 °C for 20 min, the pellet was resuspended in 400 μl SM buffer. An equal volume of chloroform (Fisher) was added, and following 30 s of vortexing, the sample was centrifuged at 2500*g* for 5 min at RT. The aqueous top layer is retained, and it was subjected to RNase I (8 U final concentration; Ambion) and DNase (20 U final concentration; TURBO DNA-free™ Kit, Invitrogen) treatment in accordance with the manufacturer’s guidelines. To isolate DNA, the samples were incubated with 20 μL of 10% SDS and 2 μL of proteinase K (Sigma, 20 mg/mL) for 20 min at 56 °C, prior to lysis by the addition of 100 μL of phage lysis buffer (4.5 M guanidine thiocyanate, 45 mM sodium citrate; 250 mM sodium lauroyl sarcosinate; 562.5 mM β-mercaptoethanol; pH 7.0) with incubation at 65 °C for 10 min. Viral DNA was purified by two treatments with an equal volume of phenol to chloroform to isoamyl alcohol (25:24:1) and passing the resulting purified DNA through a QIAGEN Blood and Tissue Purification Kit and eluting samples in 40 μL of AE buffer (Qiagen). The DNA concentrations were equalised prior to amplification using an Illustra GenomiPhi V2 kit (GE Healthcare). Amplifications of purified viral DNA was performed in triplicate on all samples as described by the manufacturer for a period of 16 h. Subsequently, products from each of the triplicate reactions and 12 μL of each corresponding original viral DNA purification were pooled together and used for paired-end Nextera XT library preparation (Illumina). All samples were sequenced on an Illumina HiSeq at GATC in Germany. Raw sequence data generated as part of this research has been deposited in the Sequence Read Archive under BioProject PRJNA446038.

### Analysis of virome sequencing data

The quality of the raw reads was visualised with FastQC v0.11.3 [33]. Nextera adapters were removed with cutadapt v1.9.1 [34], followed by read trimming and filtering with Trimmomatic v0.36 [35] to ensure a minimum length of 60, maximum length of 250, and a sliding window that cuts a read once the average quality in a window size of four falls below a Phred score of 30. Reads were classified against the Non-Redundant (nr) database at NCBI as of December 4, 2017, using the Kaiju classifier [15] which classifies based on amino acid sequence similarity. Reads were also assembled on a per sample basis with the metaSPAdes assembler [36]. Redundancy between samples was removed by aligning all contigs against each other with BLASTn as implemented in BLAST+ v2.2.28 [37] keeping the longer contig, where a hit was counted as at least 90% identity over 90% of their length. In order for a contig to be included in the final analysis, it must be detected as viral by Virsorter [12] in the virome decontamination mode, or be at least 1 kb in length and contain a gene from one known Prokaryotic Virus Orthologous Group (pVOG) [38] as detected by HMMscan (full sequence *E* value cutoff of 1-e05) as implemented in HMMER v3.1b1 [39], or contain at least one gene from a known human virus as detected by BLASTp as implemented BLAST+ v2.2.28 [37] against human viruses in the RefSeq database (*E* value cutoff of 1-e10) [40]. Integrase genes were detected by counting any hits to a pVOG that contains any gene annotated as an integrase. This allowed for the inclusion of known viruses, putative viruses, potential novel viruses bearing little to homology to known viruses, and partially assembled low abundance viruses. The quality filtered reads were then aligned to this contig set using bowtie2 v2.1.0 [41] using end-to-end alignment mode. A count table was generated with samtools v0.1.19 [42], which was then imported into R v3.3.1 for statistical analysis. In order for a contig to be counted as present in a sample, it needed to have at least a 25% breadth of coverage; otherwise, counts were set to zero.

Alpha diversity and beta diversity were generated using PhyloSeq v1.16.2 [43], which also was used for principal coordinate analysis as implemented in Ape v3.5. PERMANOVA as implemented in the Adonis function in the Vegan package was used for associating distance matrices with metadata factors. PERMANOVA as implemented in the Adonis function in the Vegan package was used for associating distance matrices with metadata factors. Spearman correlation between samples was calculated using the cor.test() function as implemented in R v3.3.1. Viruses were classified to a taxonomic group by the method previously utilised by Zuo et al. [8]. Briefly, amino acid sequences from open read frames (ORFs) predicted by Prodigal v2.6.3 [44] were matched against the UniProt TrEMBL database as of December 4, 2017. Taxonomy was then assigned to each contig based on the most abundant taxa detected by protein BLAST. All statistical analysis was performed in R v3.3.1 throughout this study.

## Additional files

Additional file 1: **Table S1.** Metadata and sample timeline for donors and recipients. Donor and patient demographics are detailed as are the faecal sampling time points (a tick denoting sample collection). In the case of donors, additional samples were collected in order to correlate with recipient timelines. This is an amended table from the study of Jalanka et al. [4] in which the bacteriome in these individuals was examined. (XLSX 11 kb)

Additional file 2: **Figure S1.** Viral alpha diversity. Alpha diversity estimates per time point for the recipients compared with the donors for (A) viral richness (Chao1) and (B) diversity (Shannon) are represented using boxplots. Whiskers indicate the highest or lowest occurring value within 1.5*IQR (interquartile range) of the upper or lower quartile. Paired Wilcoxon rank sum test, *p* ≤ 0.05 (*). (PDF 63 kb)

## Abbreviations

FMT: Faecal microbiota transplantation
rCDI: Recurrent *Clostridium difficile* infection
UC: Ulcerative colitis
