# Supplementary figures and images for: Long-term colonisation with donor bacteriophages following successful faecal microbial transplantation
Source: Microbiome. 2018 Dec 10;6:220. doi: 10.1186/s40168-018-0598-x (PMC6288847; doi:10.1186/s40168-018-0598-x)

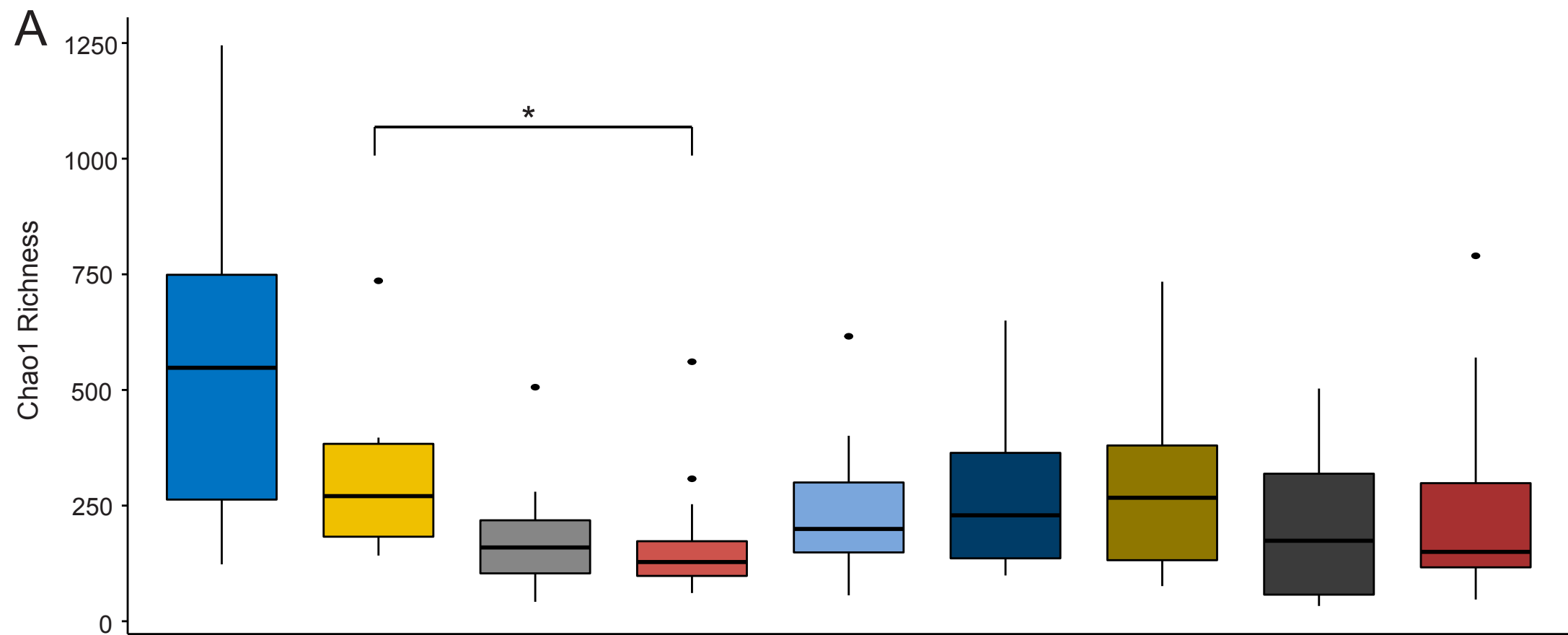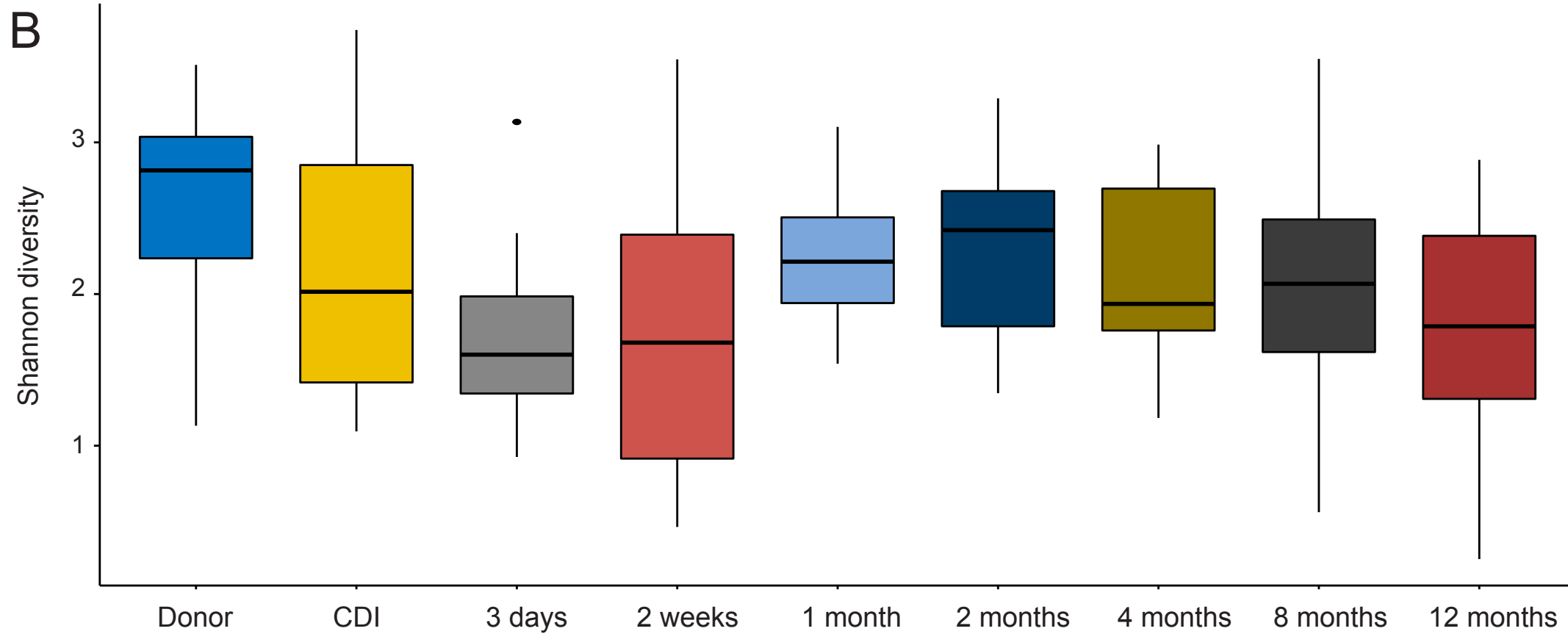

Supplement: Supplementary file 2 — Figure S1. Viral alpha diversity. Alpha diversity estimates per time point for the recipients compared with the donors for (A) viral richness (Chao1) and (B) diversity (Shannon) are represented using boxplots. Whiskers indicate the highest or lowest occurring value within 1.5*IQR (interquartile range) of the upper or lower quartile. Paired Wilcoxon rank sum test, p ≤ 0.05 (*). (PDF 63 kb) [file 40168_2018_598_MOESM2_ESM.pdf]
